# Acylation of Anisole With Benzoyl Chloride Over Rapidly Synthesized Fly Ash–Based HBEA Zeolite

**DOI:** 10.3389/fchem.2021.683125

**Published:** 2021-06-18

**Authors:** Alechine E. Ameh, Nicholas M. Musyoka, Oluwaseun Oyekola, Benoit Louis, Leslie F. Petrik

**Affiliations:** ^1^ Environmental and Nano Science Research Group, Department of Chemistry, University of the Western Cape, Bellville, South Africa; ^2^ Centre for Nanostructures and Advanced Materials (CeNAM), Chemicals Cluster, Council for Scientific and Industrial Research (CSIR), Pretoria, South Africa; ^3^ Department of Chemical Engineering, Cape Peninsula University of Technology, Cape Town, South Africa; ^4^ Institut de Chimie et Procédés pour l’Energie l’Environnement et la Santé (ICPEES), UMR 7515, CNRS, Université de Strasbourg, Strasbourg, France

**Keywords:** coal fly ash, HBEA zeolite, molar composition, acylation reaction, conversion, selectivity

## Abstract

Stable HBEA zeolite with high surface area and strong acid sites was synthesized from coal fly ash–based silica extract via indirect hydrothermal synthesis. The rapid HBEA hydrothermal crystallization times of 8, 10, and 12 h were achieved through a reduced molar water fraction in the synthesis composition. The HBEA zeolites prepared from fly ash silica extract exhibited well-defined spheroidal-shaped crystal morphology with uniform particle sizes of 192, 190, or 239 nm obtained after 8, 10, or 12 h of synthesis time, respectively. The high surface area and the microporous area of 702 and 722 m^2^/g were achieved as a function of shorter hydrothermal synthesis durations (10 and 24 h, respectively) compared to 48 or 72 h, which resulted in HBEA zeolites with lower surface areas of 538 and 670 m^2^/g. Likewise, temperature-programmed desorption measurements of fly ash–based HBEA zeolites revealed the presence of weak and strong acid sites in the zeolite. The submicron crystal sizes with a well-defined porosity of HBEA zeolites enhanced the diffusion of anisole and benzoyl chloride molecules toward the active acid sites and hence showed better conversion and selectivity in acylation products. High conversion of benzoyl chloride with anisole was achieved, reaching up to 83% with a 93–96% selectivity toward 4-methoxyacetophenone.

## Introduction

Liquid-phase catalytic reactions producing aromatic ketones, as prominent intermediates or final products, are used to prepare high-added-value molecules for applications in fine chemicals, pharmaceuticals, agrochemicals (pesticides and plasticizers), flavors, and dyes. Conventionally, these aromatic ketones have been produced using the Friedel–Crafts acylation reaction, catalyzed with homogeneous catalysts, these being either Lewis acids (AlCl_3_, TiCl_4_, FeCl_3_, InCl_3_, or BF_3_) or strong protonic acids such as concentrated H_2_SO_4_, H_3_PO_4_, HCl, or HNO_3_ (Mu et al., 2015; Wang et al., 2017). Unfortunately, these efficient transformations generate environmentally corrosive and highly toxic wastes in the process, which render the recovery and regeneration of the catalyst difficult (Chen and Ko, 2012). To overcome these challenges, extensive research focused on the development of eco-friendly heterogeneous acid catalysts that can be efficiently reused has been carried out. Such catalysts include zeolites (or metal-based zeolites), clays, Nafion-H, and mesoporous silica–alumina (Vinu et al., 2005; Mu et al., 2015).

The shape selectivity of micro- and mesoporous zeolite catalysts significantly prevents the generation of undesired by-products, which is an advantage over other solid catalysts in Friedel–Crafts reactions (Salavati-Niasari et al., 2004; Wang et al., 2017). In addition, the design of zeolite structures with tailored properties allows the achievement of an efficient performance through the ability to produce either micron- or nano-size crystals with Å-sized microporosity. These changes promote the reduction of diffusion paths within the zeolite frame while ensuring a larger surface area of the zeolite (Tosheva and Valtchev, 2005; Ding et al., 2009). Furthermore, the tailoring of zeolite porosity can allow enhanced pore size distribution, thereby inducing a hierarchical structure with both micro- and mesopores present within the zeolite. The combination of elevated specific surface area, appropriate pore size, and pore cavities favors the accessibility of molecules within and outside the zeolite structure (Verboekend et al., 2016; Zhang and Ostraat, 2016; Al-Eid et al., 2019).

The International Zeolite Association (http://www.iza-online.org/) has reported more than 240 different zeolites. Among those different zeolite structures, extensive research and industrial applications have been particularly devoted to the following big five zeolites: MOR, FAU, BEA, FER, and MFI (Yilmaz et al., Müller, 2012). Apparently, this is due to the open-pore structure of their three-dimensional 12-membered ring interconnected channels. The BEA zeolite became a catalyst of choice in numerous Friedel–Crafts alkylation and acylation reactions (Derouane et al., 2000; Sartori and Maggi, 2006; Chen et al., 2015). Also, the high catalytic activities achieved over the BEA zeolite were strongly related to the presence of numerous defects in the framework (Gabrienko et al., 2010). Typically, the nature, purity, and reactivity of the silica and alumina sources are important to guarantee high zeolite phase purity and thus, high catalytic performance (Losch et al., 2016). Unfortunately, the preparation of BEA zeolites using pure synthetic silica and alumina precursors involves high manufacturing costs of silica and alumina and templating agents (Vichaphund et al., 2019). There is, therefore, a need to find alternative, cheap, and sustainable silica and alumina sources.

Various natural products such as clay and waste by-products (Ma et al., 2014; Maia et al., 2015), including siliceous minerals (Liu et al., 2015), biomass of plant ash residues (Belviso, 2018), electronic waste (Mallapur and Oubagaranadin, 2017), and coal fly ash (CFA) (Ameh et al., 2017; Ameh et al., 2020a), have been used as feedstock for the synthesis of zeolites.

The advantage of CFA is based on its negligible cost, abundance, availability, and the suitable content of silica and alumina that allows its complete conversion into zeolites (Wdowin et al., 2014). Likewise, the framework structure of typical zeolites consists of aluminosilicate building blocks (Martinez Sanchez and Pérez Pariente, 2011), which are similar to the silica and aluminum components in CFA (Musyoka et al., 2011; Boycheva et al., 2013; Ameh et al., 2017). Interestingly, Ameh et al. (2020a) and Petrik et al. (2020) have demonstrated that CFA can act as an inexpensive and recyclable feedstock for the production of pure BEA zeolite phase via an indirect hydrothermal route. Recently, pure phase BEA zeolite was crystallized from CFA-based extract at a reduced hydrothermal time of 24 h (Ameh et al., 2020a). However, the hydrothermal conversion of the gel containing silica, alumina, mineralizing agent, structure-directing agent, and water at a temperature of 140°C required a long crystallization time until complete phase formation was achieved.

In the present study, CFA was utilized as a suitable feedstock for the formation of the BEA zeolite. Herein, the influence of the molar water content in the molar regime used as the synthesis precursor gel and its impact on the reduced hydrothermal time and phase purity were investigated. The study also aimed to establish the role of the physicochemical properties of HBEA zeolites upon their activity and selectivity in the Friedel–Crafts acylation of anisole.

## Experimental Design

### Extraction of Nanosilica

Ameh et al. (2020a) gave a detailed description of nanosilica extraction from CFA. At a mass ratio of 1:1.2, CFA and NaOH pellets (97%, Kimix) were weighed and poured into a porcelain crucible, which was then transferred to a muffle furnace set at 550°C for 1.5 h. After cooling, the fused alkaline material was ground into a fine powder using a lab-scale ball mill and dissolved in deionized water (1:5 solid/liquid ratio w/v). The obtained filtrate was then stored until it was needed for the extraction process.

Subsequently, concentrated sulfuric acid (95–99%, Sigma) was added dropwise to the filtrate until a white precipitate was formed. The white precipitate was dried at 70°C overnight and was then heated under reflux with 1.5 M oxalic acid (99%, Sigma) at 80°C for 6 h in a solid/liquid ratio of 10:1 w/v. The silica extract was recovered by hot filtration, and the solid fraction was then dried overnight at 70°C.

### Synthesis of BEA Zeolite

The experimental design followed a one-step-at-a-time synthesis protocol as described in Supplementary Table S1. Different molar formulations were prepared by adding 1.91 g of dried aluminosilica extract to 0.10 g NaOH diluted in a given (*x*) amount of deionized water. 4.24 g tetraethylammonium hydroxide (TEAOH) was then added carefully to generate the molar composition of 1.0 Si: 0.017 Al: 0.241 Na: 0.399 TEAOH: *x* H_2_O, where *x* is 1.776, 2.661, 3.991, 5.986, or 8.980 (Supplementary Table S1). The resulting reaction mixture was aged for 30 min at room temperature and then transferred into a 100-ml-capacity Teflon-lined stainless steel pressure vessel. Afterward, the reaction vessel was heated hydrothermally at a set temperature of 140°C for durations of 8, 10, 12, 24, 48, or 72 h under static conditions.

The pressure vessel was allowed to cool down, and then the recovered solid particles were repeatedly washed with deionized water and filtered. The resultant mass of as-synthesized products was determined after drying the solids overnight at 70°C. Thereafter, the products were detemplated in air at a ramping rate of 2°/min for 2 h at 120°C and then at 550°C for 3 h at a ramping rate of 3°/min to remove the TEAOH. Each sample was ion-exchanged with 1 M ammonium nitrate (NH_4_NO_3_) solution in a mass/volume ratio of 1 g:50 ml (zeolite/NH_4_NO_3_) at 80°C for 2 h under stirring. Upon completion, the recovered zeolite was oven-dried at 80°C overnight and thereafter, calcined in air at 200°C for 2 h and then at 500°C for 4 h (ramp 10°/min) to remove NH_3_ and produce the HBEA zeolite form.

### Characterization

The morphology and crystal size of the samples were analyzed using a Zeiss Gemini Auriga high-resolution scanning electron microanalyzer (HR-SEM) equipped with a CDU-lead detector at 25 kV and a tungsten filament. Transmission electron microscopy (TEM) images were obtained using a field-emission gun FEI Teenai G2 20 instrument operating at 200 kV.

The phase purity and crystal structure of the raw materials and products were determined via X-ray diffraction (XRD) patterns on a Philips X-pert pro-MPD X-ray diffractometer using Cu–K radiation, operated at 40 kV and 40 mA to analyze and collect diffractograms in the range of 5–60° 2θ with a step size of 0.02°/s. The crystalline mineral phases were identified by performing a match search and comparing the patterns with the database of standard peak patterns provided by the International Centre for Diffraction Data (ICDD), using High score Xpert software. The relative crystallinity of the HBEA zeolite samples (H8, H10, H12, H24, H48, and H72) was estimated by analyzing the peak areas at 7.7 and 22.5° 2θ.

^27^Al single-pulse MAS NMR experiments were performed at a magnetic field of 11.4 T on a spectrometer with the corresponding Larmor frequencies of 130.3 (^27^Al) and 99.3 (^29^Si) MHz. All single-pulse spectra were acquired using a single pulse at 90° with a recovery delay of 0.5 s (^27^Al) or 25 s (^29^Si). The spectra were accumulated during 1,200 scans (^27^Al) or 300 scans (^29^Si) using a 4 mm BBO probe at a spinning rate of 14 kHz (^27^Al) and 8 kHz (^29^Si) for all experiments. The samples were packed into standard zirconia 4 mm rotors (Bruker).

The textural properties of HBEA zeolites and the BET surface area were acquired using a Micromeritics ASAP 2020 HD analyzer at 77 K. The N_2_ adsorption/desorption isotherms were evaluated by subjecting the samples to degassing down to 10^–7^ bar at 200°C for 8 h to remove moisture and/or other volatile residues. The microporous and mesoporous surface areas and the pore volume were calculated using the Brunauer–Emmett–Teller (BET) model in the region of 0.01< P/P_o_ <0.25, and t-plot measurements were used to differentiate between the external surface area and the microporous area. The density functional theory (DFT) model was used to obtain the pore size distributions, basing it on the cylindrical pore geometry, whereas the total pore volume was determined using the Horvath–Kawazoe (*HK*) model at P/P_o_ ∼0.99.

Ammonia temperature-programmed desorption (NH_3_-TPD) was carried out using a Micromeritics Autochem II 2920 chemisorption analyzer. Approximately 30–50 mg of the HBEA zeolites was pretreated at 550°C (heating ramp: 10°/min) in 10 ml/min He flow for 1 h to desorb moisture. Afterward, the zeolite samples were cooled to 50°C, and ammonia was adsorbed for 30 min using 20 ml/min of 10 vol% NH_3_ in He. Each sample was then purged in flowing He for 30 min, and the NH_3_ desorption was recorded by heating the zeolite to 700°C using a 10°/min ramp. The obtained curves were normalized according to the mass of the sample, and the peak areas were analyzed using the Gauss line shape in OriginPro 8.5 software.

### Catalytic Reactions

The acylation of anisole with benzoyl chloride in this study was adapted from the work of El Berrichi et al. (2005). Prior to the reaction, 1.37 g (0.013 mol) of anisole was quickly transferred into a pressure tube held in an oil bath and purged with nitrogen gas to remove air under vigorous stirring. Thereafter, 0.07 g of the freshly calcined HBEA zeolite was added into the tube under continuous stirring at 650 rpm until a temperature of 120°C was reached. Subsequently, 0.55 g (0.004 mol) of benzoyl chloride (acylating agent) was added to the mixture contained in the pressurized vessel.

Samples were collected at a specific reaction time and were cooled and quickly filtered for further analysis. Gas chromatography was used to analyze, identify, and quantify final and intermediate products. Each of the components was separated in an Agilent 7890A GC equipped with a DB-EUPAH capillary column (20 m × 0.18 mm, film thickness 0.14 µm) oven programmed for a 2 min hold period at 70°C, followed by heating (15°C/min H gas) to 250°C, after which the temperature was held constant for 14 min. The injector temperature was 250°C, and the injector split ratio was set to 100:1 (100 ml/min He gas). The GC was equipped with a flame ionization detector (FID).

The influences of the reaction duration, aluminum content, and textural properties of HBEA zeolites on the acylation reaction were explored. The degrees of conversion, selectivity, and product yield were determined by considering the amount of reactants and products at the end of each reaction cycle using the following equations:

$$\text{\%}\;\text{Conversion}=\left[\frac{{\text{P}}_{2\text{Me}}+{\text{P}}_{4\text{Me}}+{\text{P}}_{\text{PHB}}}{{\text{P}}_{2\text{Me}}+{\text{P}}_{4\text{Me}}+{\text{P}}_{\text{PHB}}+P_{BzCl}}\right]\times 100,$$ (1)

$$\text{\%}\;\text{Selectivity}=\left[\frac{{\text{P}}_{2\text{Me}}\;\text{or}\;{\text{P}}_{4\text{Me}}\;\text{or}\;{\text{P}}_{\text{PHB}}}{{\text{P}}_{2\text{Me}}+{\text{P}}_{4\text{Me}}+{\text{P}}_{\text{PHB}}}\right]\times 100,$$ (2)

$$\text{\%}\;{\text{Yield}}_{i}=\left[\frac{\text{conversion}\times \text{selectivity }}{100}\right].$$ (3)

P_2Me_, P_4Me_, and P_PHB_ were the quantities of the products (2-methoxybenzophenone, 4-methoxybenzophenone, and phenyl benzoate, respectively) at a specific time, and P_BzCl_ was the quantity of benzoyl chloride.

## Results and Discussion

### Extracted Nanosilica

**FIGURE 1:**
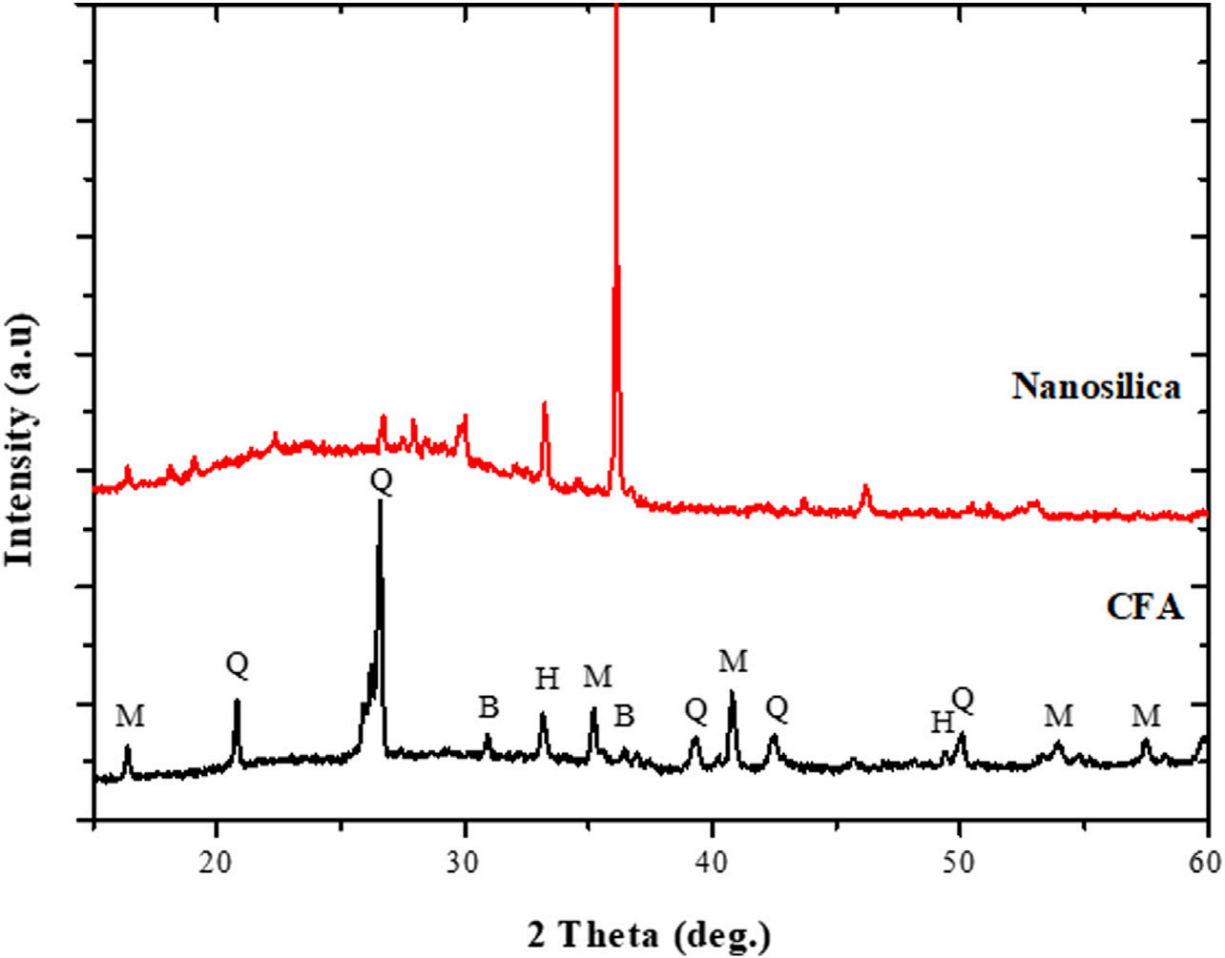
XRD patterns of raw CFA and extracted nanosilica.

The X-ray diffraction patterns of the CFA feedstock and nanosilica extract are presented in Figure 1. The raw CFA was predominantly composed of two major phases, mullite (M) and quartz (Q), with a glassy amorphous phase between 20 and 40° 2θ (Figure 1). Other mineral phases observed in the CFA include hematite (H) and magnetite (B). After the extraction process involving fusion, precipitation, and oxalic treatment, the mineral phases present in the CFA were completely transformed into amorphous materials that exhibited a broad hump between 15 and 37° 2θ, typically characteristic of nanosilica particles (Figure 1). Besides, the remaining diffractions at 16 and 33° 2θ, and their shape, observed in the nanosilica extract could be associated with aluminosilicate glass (3Al_2_O_3_.SiO_2_) or crystalline nanosilica (Yuvakkumar et al., 2012; Ameh et al., 2020a).

### Hydrothermal Synthesis of BEA Zeolites From Nanosilica Extract

**FIGURE 2:**
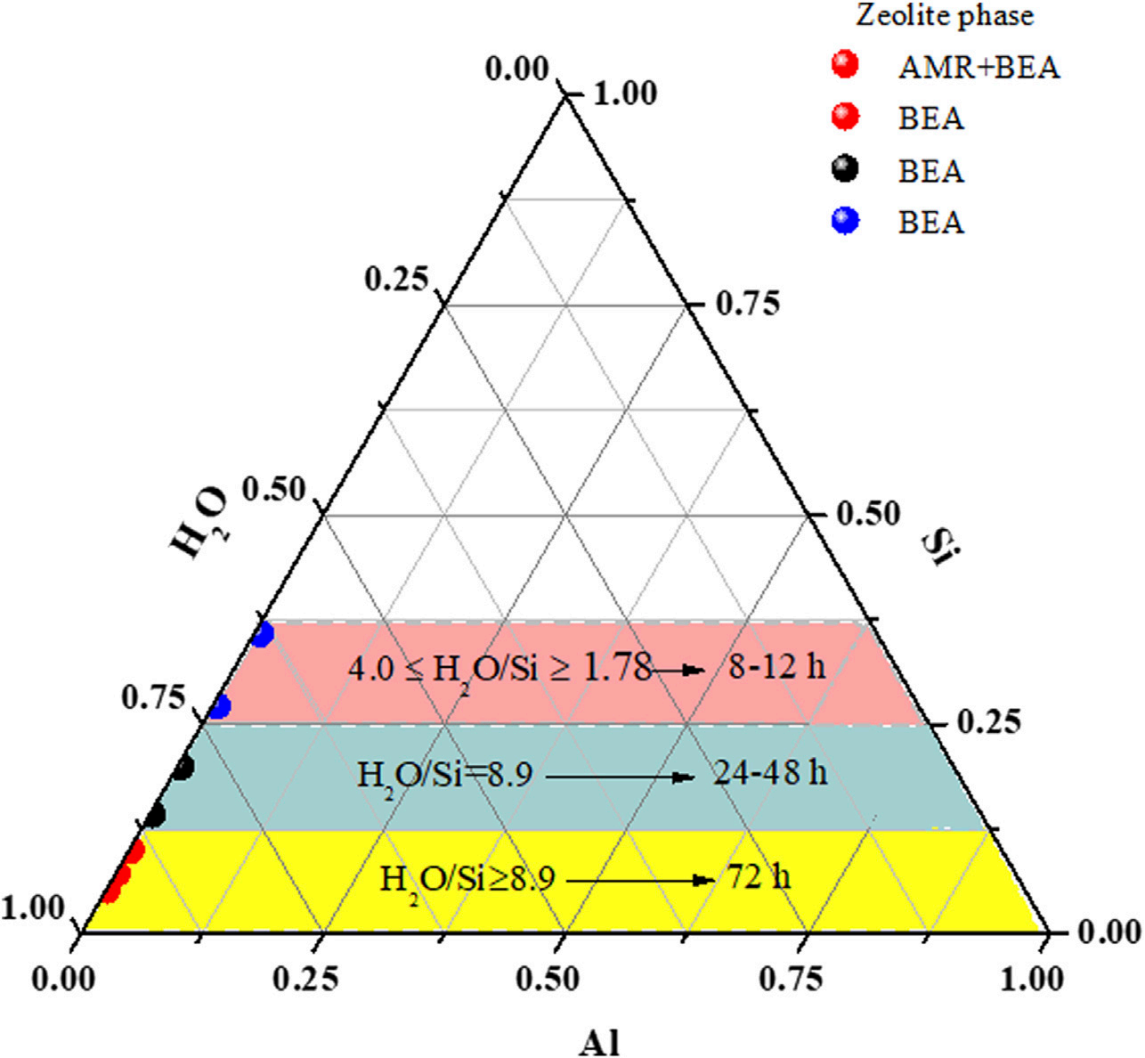
Ternary diagram representing rapid phase formation as a function of reduced molar H_2_O. *AMR = amorphous.

Figure 2 and Supplementary Figure S1 depict the effect of reduced molar quantities of water in the synthesis mixture on the hydrothermal time and phase purity of BEA zeolites. The XRD pattern in Supplementary Figure S1 only shows the formation of amorphous material when the molar H_2_O fraction was 17.958 (H01) after 72 h of hydrothermal treatment. Two major associated diffractions at 7.7 and 22.4°, which correspond to (101) and (302) reflections, respectively, appeared as the molar H_2_O fraction was reduced to 12.828 (H02). This suggests that the H02 sample contained both the BEA zeolite and amorphous material. With a further reduction of the H_2_O molar fraction (H72 = 8.980), the main diffraction peaks at 7.7, 13.4, 22.4, 27.1, 28.7, 29.6, and 43.4° of a typical BEA zeolite were observed (Database of Zeolite Structure, http://www.iza-structure.org/databases/). This confirms that a decrease in the water molar fraction from 17.958 to 8.980 allowed a shift from the amorphous phase to BEA crystal formation. This is similar to the findings of Manrique et al. (2016) and Gabrienko et al. (2010), who showed that a higher H_2_O/SiO_2_ ratio led to the formation of amorphous materials rather than a pure BEA phase. Similarly, with the molar H_2_O = 8.980, the BEA framework structure was maintained when the hydrothermal time was reduced to 24 and 48 h (H24 and H48, respectively) (Figure 2, Supplementary Figure S1).

Herein, the molar fraction of H_2_O was further reduced under the hydrothermal condition of 140°C for 12 h. According to the XRD patterns (Figure 2), two main diffraction lines were detected when the molar fraction of H_2_O was 5.99 (C11). In this case, the broad diffraction between 10 and 11° 2θ could be associated with the presence of amorphous materials, while the second peak at 22.5° 2θ tends to indicate the appearance of the BEA zeolite phase. As the molar fraction of H_2_O was further diminished to 3.99, 2.66, or 1.78, all the diffraction peaks of a fully crystalline BEA zeolite were observed (C12, C13, and C14). This further confirms that a low molar fraction of H_2_O (between 3.99 and 1.78) induces the complete and rapid formation of pure BEA zeolites under the applied conditions, hence eliminating the presence of amorphous materials (Figure 2, Supplementary Figure S1).

With the molar fraction of H_2_O = 3.99, 2.66, or 1.78, the gel was treated hydrothermally at different times—8 or 10 h—at 140°C (Supplementary Figure S1). After 10 h, the diffraction patterns of samples B10, B11, and B12 matched the characteristic BEA zeolite structure perfectly (Figure 2, Supplementary Figure S1) as reported by the Database of Zeolite Structure (http://www.iza-structure.org/databases/). On the other hand, after 8 h of hydrothermal synthesis, only the sample with a 2.66 (A8) molar fraction of H_2_O exhibited all the characteristic diffraction of BEA zeolites. It was found that the peak intensity at 7.7 and 22.4° decreased with 1.78 and 3.99 molar fractions of H_2_O (A7 and A9, respectively). However, the other associated BEA zeolite peaks at 13.4, 27.1, 28.7, and 43.4° thus gradually disappeared with a decrease or increase in the molar fraction of H_2_O (Supplementary Figure S1). This indicates that a 2.66 molar fraction of H_2_O is the boundary condition for the BEA zeolite formation at 140°C for 8 h. Hence, pure BEA zeolites could be synthesized from the low molar water fraction of 1.78 after a short time of 8 h, with some residual amorphous phase (Figure 2). As the water content was reduced in the synthesis mixture, the solution becomes supersaturated, thereby enhancing the nucleation process. Meanwhile, an increase in the water content dilutes the concentration of primary aluminum and silicon species; this may hinder the induction process and lead to slow nucleation (Gabrienko et al., 2010; Manrique et al., 2016).

### Physicochemical Properties of HBEA Zeolite

The selected HBEA zeolites formed at the hydrothermal synthesis times of 8, 10, 12, 24, 48, and 72 h (A8, B10, C12, H24, H48, and H72) were exchanged into their respective H-forms (H8, H10, H12, H24, H48, and H72, respectively) prior to in-depth characterization.

**FIGURE 3:**
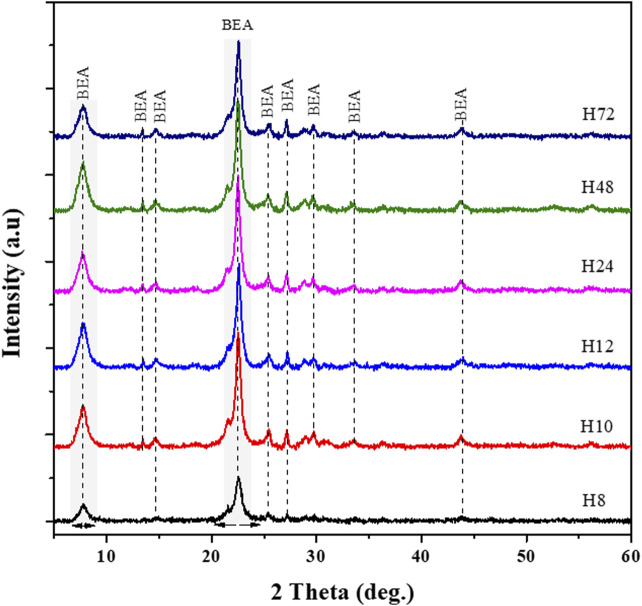
XRD patterns of as-synthesized HBEA zeolites produced from CFA nanosilica extract at different water contents and hydrothermal times.

The XRD patterns of the six selected HBEA zeolite samples are compared in Figure 3. All the diffraction patterns display characteristic peaks, which can undoubtedly be attributed to the HBEA zeolite phase. However, sample H8 showed a lower intensity of the two main peaks at 2θ = 7.7, and 22.4° and four minor diffractions at 2θ = 13.4, 27.1, 28.7, and 29.6° (Figure 3). The increasing peak intensity of the main peaks and the reduced background scattering of the other HBEA samples are indicative of highly crystalline products. The relative crystallinity showed no specific trend but indicated that crystalline HBEA zeolites (>70%) were prepared under the applied conditions, except the H8 zeolite, which remained below 45%. Besides, the short hydrothermal times of 10 and 12 h enhanced the high relative crystallinity of samples H10 and H12 to above 90% compared to the 79–88% crystallinity obtained after longer synthesis durations (H24, H48, and H72, respectively). These results validate the fact that low molar fraction of water promoted supersaturation, thus enhancing the mobility of the reacting species in the synthesis mixture, and, by close contact, promoted a rapid nucleation rate.

**FIGURE 4:**
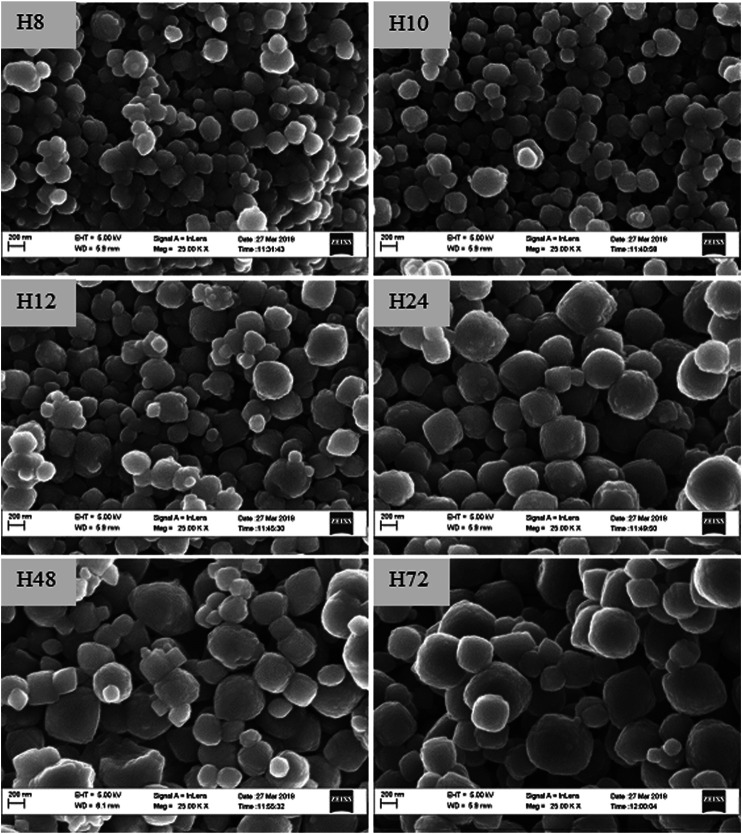
SEM images of as-synthesized HBEA zeolites produced from CFA nanosilica extract at different water contents and hydrothermal times.

**FIGURE 5:**
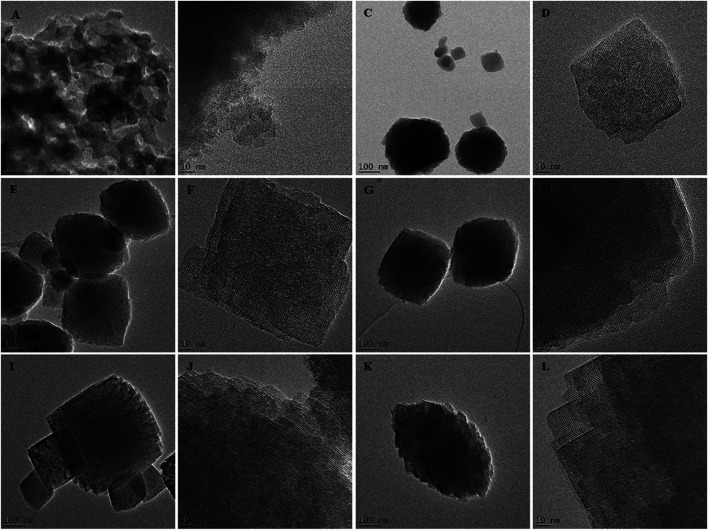
TEM images of the as-synthesized HBEA zeolites H8 **(A,B),** H10 **(C,D),** H12 (**E,F),** H24 **(G,H),** H48 **(I,J),** and H72 **(K,L)** produced from CFA nanosilica extract at different water contents and hydrothermal times.

To validate those findings, the morphology of as-synthesized crystals was compared using SEM-EDS and HRTEM analysis, as presented in Figures 4, 5.

The SEM micrographs (Figure 4) show that the H8, H10, H12, H24, H48, and H72 (8, 10, 12, 24, 48, and 72 h, respectively) samples exhibit a spheroidal-shaped morphology. Such a spheroidal structure is typical of BEA zeolites (Zhang et al., 2016; Ameh et al., 2020a). Si, O, and Al species represented the main elemental content of the CFA-derived zeolite framework of the HBEA crystals, as presented in Table 1. Likewise, at low TEM magnification, Figure 5 confirms a similar spheroidal zeolite HBEA shape, which is in agreement with SEM micrographs (Figure 4). The as-synthesized samples exhibit a continuous network of microporous and mesoporous channels present in the HBEA framework, with crystal sizes between 50 and 400 nm. In sample H8 (Figures 5A,B), small irregular-shaped crystals which consisted of disordered mesopores were observed (Huang et al., 2017). The H10 and H12 samples exhibited an assembly of aggregated nanocrystals with lattice fringes aligned in different directions (Figures 5C–F). In contrast, Figures 5G–L (H24, H48, and H72) highlight the formation of polycrystals with intergrown nanocrystallites of bigger sizes ranging between 100 and 400 nm and lattice fringes aligned in the same direction. The lattice fringes reveal a distinguishable plane with about 1.12 nm of d-space, which corresponds to the (101) direction as observed in Supplementary Figure S4. This indicated the coexistence of micropores and mesopores in the synthesized BEA zeolite.

**TABLE 1:** Elemental composition and crystal size of HBEA zeolites obtained using SEM-EDS.

Sample	O	Al	Si	Si/Al ratio^a^	^b^Crystal size (nm)
H8	54.2	1.5	44.3	30	192
H10	62.0	1.5	36.5	25	190
H12	62.5	1.4	36.1	25	239
H24	61.3	1.6	37.1	24	370
H48	58.6	1.5	39.9	26	321
H72	65.5	1.4	33.0	23	379

a The elemental composition and Si/Al ratio were determined from SEM-EDS.

b The crystal size was determined from SEM using ImageJ software.

The uptake of Al in the BEA framework was found to be consistent for all samples, as indicated by the Si/Al ratios obtained by SEM-EDS analysis. Hence, the synthesis duration did not alter the Si/Al ratios. All HBEA zeolites have 23≤ Si/Al ≤26, except for sample H8 with Si/Al ratio = 30 (Table 1). This case suggests that residual silica which was not completely crystalline remained present as amorphous material in the sample.

All the as-synthesized HBEA zeolite particles were composed of uniform particles with a size range of 190–380 nm, which gradually increased to above 300 nm with longer synthesis durations of 24, 48, and 72 h (Table 1). This observation further proves that more concentrated supersaturated mixtures are beneficial for rapid nucleation. This promotes the rapid transformation of the zeolite nuclei into well-ordered, small BEA crystals and limits the crystal growth in low–molar water fraction environments. Möller et al. (2011) reported that the early-stage nucleated zeolite crystallites are restricted from further growth due to the constant contact of the reacting species in low–molar water fraction mixtures, allowing rapid incorporation and utilization of available species in numerous nuclei.

### Framework Structure and Acidic Properties of HBEA Zeolite

**FIGURE 6:**
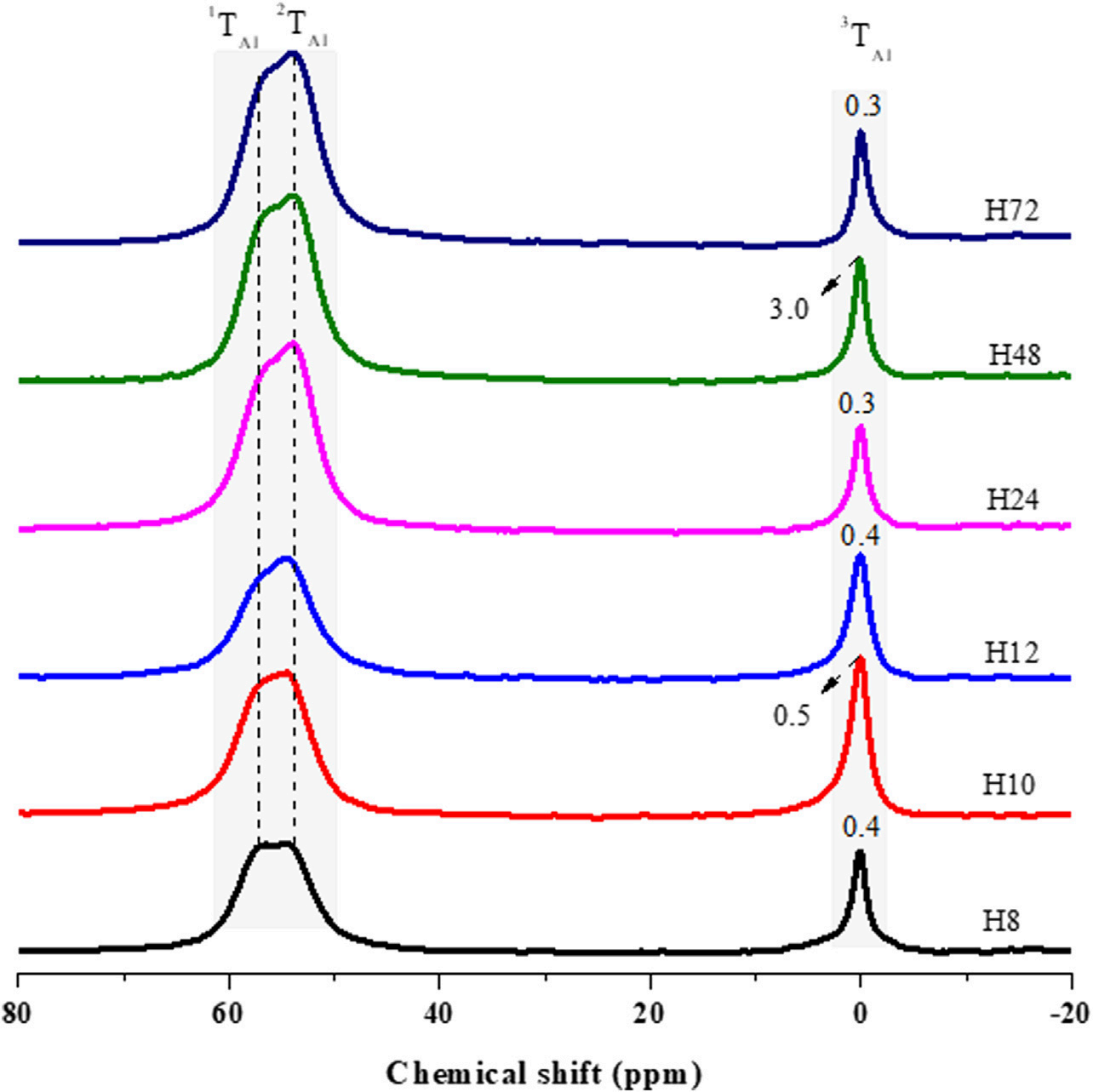
^27^Al MAS NMR analysis of as-synthesized HBEA zeolites produced from CFA nanosilica extract at different hydrothermal times.

The ^29^Si and ^27^Al MAS NMR were analyzed with a spinning speed of 8 kHz at a magnetic field strength of 11.4 T. Figure 6 presents ^27^Al MAS NMR of the H8, H10, H12, H24, H48, and H72 zeolite samples produced at the hydrothermal synthesis times of 8, 10, 12, 24, 48, and 72 h, respectively.

^27^Al MAS NMR spectroscopy was used to evaluate the environment of the Al atoms in HBEA samples (Figure 6). Two major resonance bands were observed, with the first peak appearing between δ = 60.9 and 53.8 ppm, it being attributed to tetrahedrally coordinated framework aluminum (FAl), while the second peak, appearing between δ = 0.3 and 3.0 ppm, was assigned to octahedrally coordinated extra-framework aluminum species (EFAl) (Prodinger et al., 2018; Zhao et al., 2015; Ameh et al., 2020a; Ameh et al., 2020b). To further analyze the structure, the peak position of the framework Al (FAL = ^1^T_Al_ and ^2^T_Al_) and the extra-framework Al (EFAl = ^3^T_Al_) and the peak width of EFAl were derived by deconvolution using mixed Lorentzian and Gaussian line shapes (Figure 6, Supplementary Figure S2; Supplementary Table S2).

Herein, two contributing FAl peaks of upfield (^1^T_Al_) and downfield (^2^T_Al_) resonance were resolved after deconvolution of the corresponding signal (Supplementary Figure S2; Supplementary Table S2). The upfield signal, ^1^T_Al_, occurring between 57.5 and 57.9 ppm, and the downfield signal, ^2^T_Al_, between δ = 53.9 and 54.5 ppm, were both assigned to tetrahedral Al species in HBEA zeolite samples. However, sample H48 displayed only the upfield signal ^1^T_Al_, with a high-field at δ = 60.9 ppm and a low-field at δ = 56.6 ppm (Figure 6; Supplementary Table S2). This suggests the possible presence of terminal Si-OH defect sites in the framework structure of the H48 zeolite. Manrique et al. (2016) have associated the higher upfield signal, between 60 and 63 ppm, with the presence of distorted tetrahedral Al species within the BEA framework. They further assigned the lower upfield to a regular signal of tetrahedral framework Al species.

Supplementary Figure S2 and Supplementary Table S2 clearly show that Al species were mostly incorporated into the ^2^T_Al_ tetrahedral framework (δ = 54 ppm), with only a small fraction of aluminum located in the EFAl coordination (δ = 0 ppm). The second major resonance, ^3^T_Al_, assigned to hexacoordinated extra-framework aluminum (EFAl), shows signals between δ = 0.3 and 0.5 ppm for the H8, H10, H12, H24, and H72 samples. On the other hand, sample H48 displayed a significant upward peak shift to δ = 3.0 ppm (Figure 6; Supplementary Table S2). This indicates that the signal was sensitive to the coordination environment of extra-framework Al species. The EFAl (^3^T_Al_) diminished from 3.0 to 0.3 ppm with decreasing integral intensity (H48 > H10 > H12 > H24 > H72) (Supplementary Table S2) as the Si/Al ratio increased (H48 > H10 > H12 > H24 > H72) (Table 3). During the substitution of Si by Al species, the negative charge ($SiO^{-}Al$) balanced by protons resulted in the generation of Brønsted acid sites, which can be related to framework Al atoms located in tetrahedral coordination. Furthermore, the presence of EFAl in the HBEA zeolite can be related to Lewis acid sites in the structure (Wang et al., 2017). Also, the upfield shifting of coordinated Al species in the extra-framework positions from 0 ppm to about 4 ppm signified the formation of extra-framework aluminum hydroxide species (Zhao et al., 2015). This validates the fact that the H48 sample contains distorted tetrahedral Al species due to the presence of terminal Si-OH or Al-OH sites in the structure. Moreover, the presence of tetrahedrally coordinated Al sites in all HBEA zeolites may indicate the existence of Brønsted acidity. The results presented herein further suggest that HBEA zeolites contain mostly framework Al rather than extra-framework Al, thus being potentially active catalysts due to the presence of acid sites.

**FIGURE 7:**
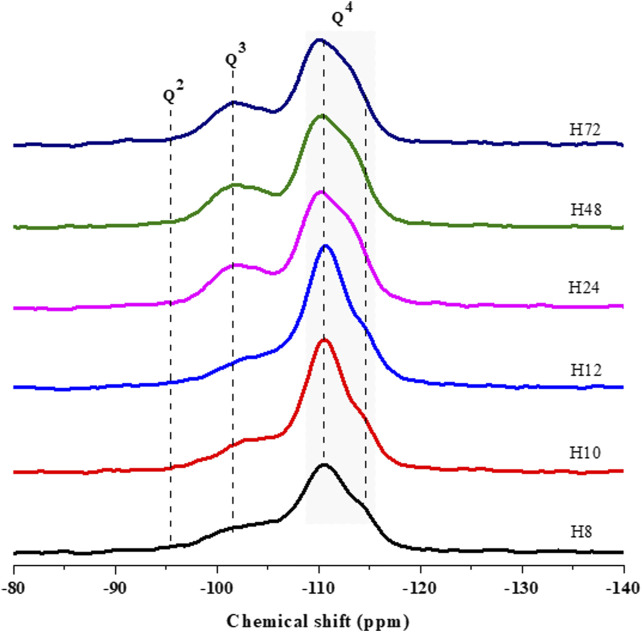
^29^Si MAS NMR spectra of HBEA zeolites produced from CFA nanosilica extract at different hydrothermal times.

Figure 7 compared the silicon nucleus environment in as-prepared HBEA zeolites by ^29^Si MAS NMR spectroscopy. The spectra elucidated two main coordination environments of Q^3^ and Q^4^ types. The chemical shift of those environments was further deconvoluted using mixed Lorentzian and Gaussian line shapes. Upon deconvolution, the framework Si/Al ratio was calculated by applying equation S1, and the influence of hydrothermal time on the peak area was evaluated (Supplementary Figure S3, Supplementary Table S3).

The resonance bands around δ = −102–104.4 ppm are assigned to the Q^3^ Si (1Al) environment. These can be related to silicon-bearing OH groups of (SiO)_3_Si(OH), whereas the resonances around δ = −110–-111 ppm are attributed to the Q^4^ Si (0Al) of four neighboring silicon atoms (after O-atoms), as shown in Figure 7. A weak shoulder at about δ = -112 and -113 ppm, may also be assigned to four coordinated atoms of related Q^4^ Si (0Al) groups. Similarly, several researchers have assigned those signals to the structural framework of BEA zeolites (Zhang et al., 2013; Zhu et al., 2017).

It is worth noting that the Q^3^ Si (1Al) of samples H10 and H24 at δ = -104 and -111 ppm shifted downward (≥0.4 ppm) to δ = -103 and -110 ppm, as seen in samples H24, H48, and H72, respectively. The difference in the Q^3^ Si (1Al) and Q^4^ Si (0Al) can be related to the degree of Si substituted by Al in the lattice of the zeolites. This can be confirmed by the decreasing framework Si/Al ratio of 30<25<24<23 as the hydrothermal time increased from 12 to 72 h due to the Al substitution process in the framework structure (H12, H24, H48, and H72, respectively) (Supplementary Table S3). Likewise, the Q^3^ area of (SiO)_3_Si(OH) of related H72 and H48 samples reached the highest amount of 26 and 27%. However, this contribution decreased to the lowest percentage of 23% (H12) as the hydrothermal time was reduced to 12 h. Relating the aforementioned observation with the shift in the Q^3^ environment, it can be noted that a short hydrothermal time promoted a reduced amount of Si-OH defect sites in the framework of CFA-based HBEA zeolites.

### Textural and Acidic Properties of HBEA Zeolite

**FIGURE 8:**
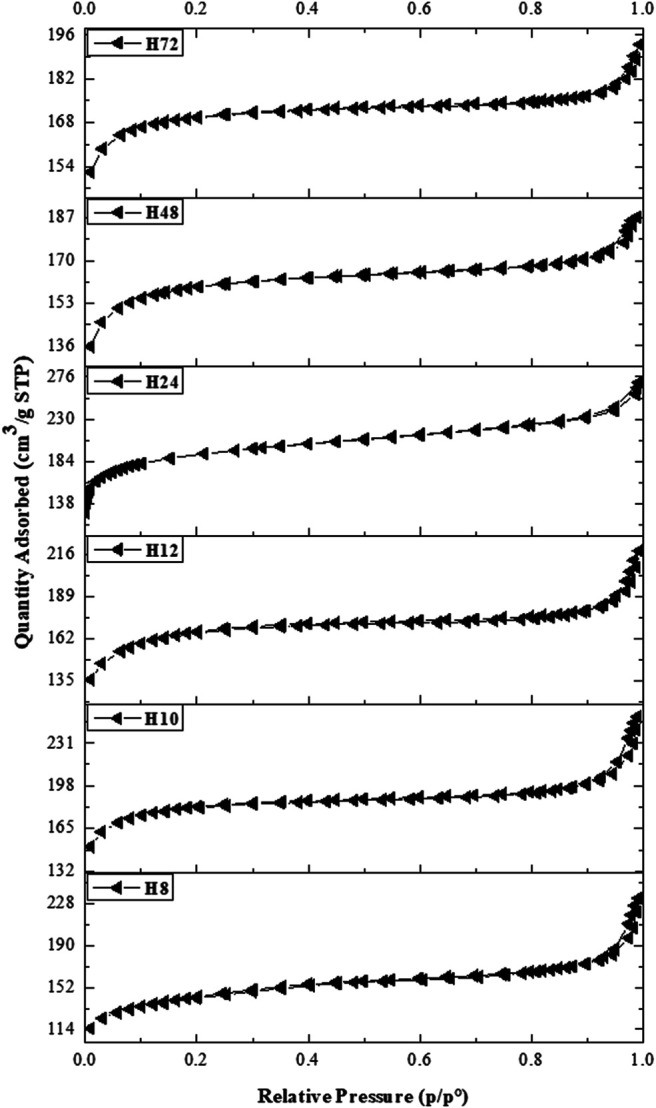
N_2_ adsorption–desorption isotherms of HBEA zeolites produced from CFA nanosilica extract at different hydrothermal times.

The N_2_ isotherms of CFA-based HBEA zeolites prepared at different hydrothermal times are presented in Figure 8. The isotherms of all samples apart from H24 and H48 exhibited both type I and type IV with small hysteresis loops indicating the presence of interconnected mesopores of the open capillary that allowed the evaporation of adsorbed nitrogen (Thommes et al., 2015). The curve profile characterizes the Langmuir adsorption of N_2_ with the corresponding micropore filling at *P/P*_*0*_ <0.17, while the type IV isotherm within the range of 0.2< *P/P*_*0*_ <0.7 is attributed to the capillary condensation within the mesopores. The combination of both micro- and mesoporous structures is a significant advantage of the CFA-based HBEA zeolite in catalysis.

Table 2 presents the textural properties of HBEA zeolites obtained after the different hydrothermal times of 8, 10, 12, 24, 48, and 72 h (H8, H10, H12, H24, H48, and H72, respectively). All HBEA zeolites possess high surface areas between 538 and 722 m^2^/g. With decreased hydrothermal time, the micropore area diminished, while the mesoporous surface area increased in some cases. These results are an indication that an acceptable degree of microporosity and a high degree of mesoporosity of the crystalline CFA-based HBEA zeolite with high surface area were obtained at the short hydrothermal times of 10–12 h.

**TABLE 2:** Textural properties of as-synthesized HBEA zeolites at different hydrothermal times.

Zeolite	S_BET_ [m^2^/g]	S_micro_ [m^2^/g]	S_meso_ [m^2^/g]	V_micro_ [cm^3^/g]
H8	538	361	177	0.12
H10	702	488	214	0.17
H12	633	425	208	0.15
H24	722	512	210	0.38
H48	538	439	99	0.20
H72	670	543	127	0.20

S_BET_: BET surface area.

S_micro_: micropore surface area was determined using the t-plot method.

S_meso_: mesopore surface area = S_BET_ - S_micro._

V_micro_: micropore volume was determined using the BJH method.

**FIGURE 9:**
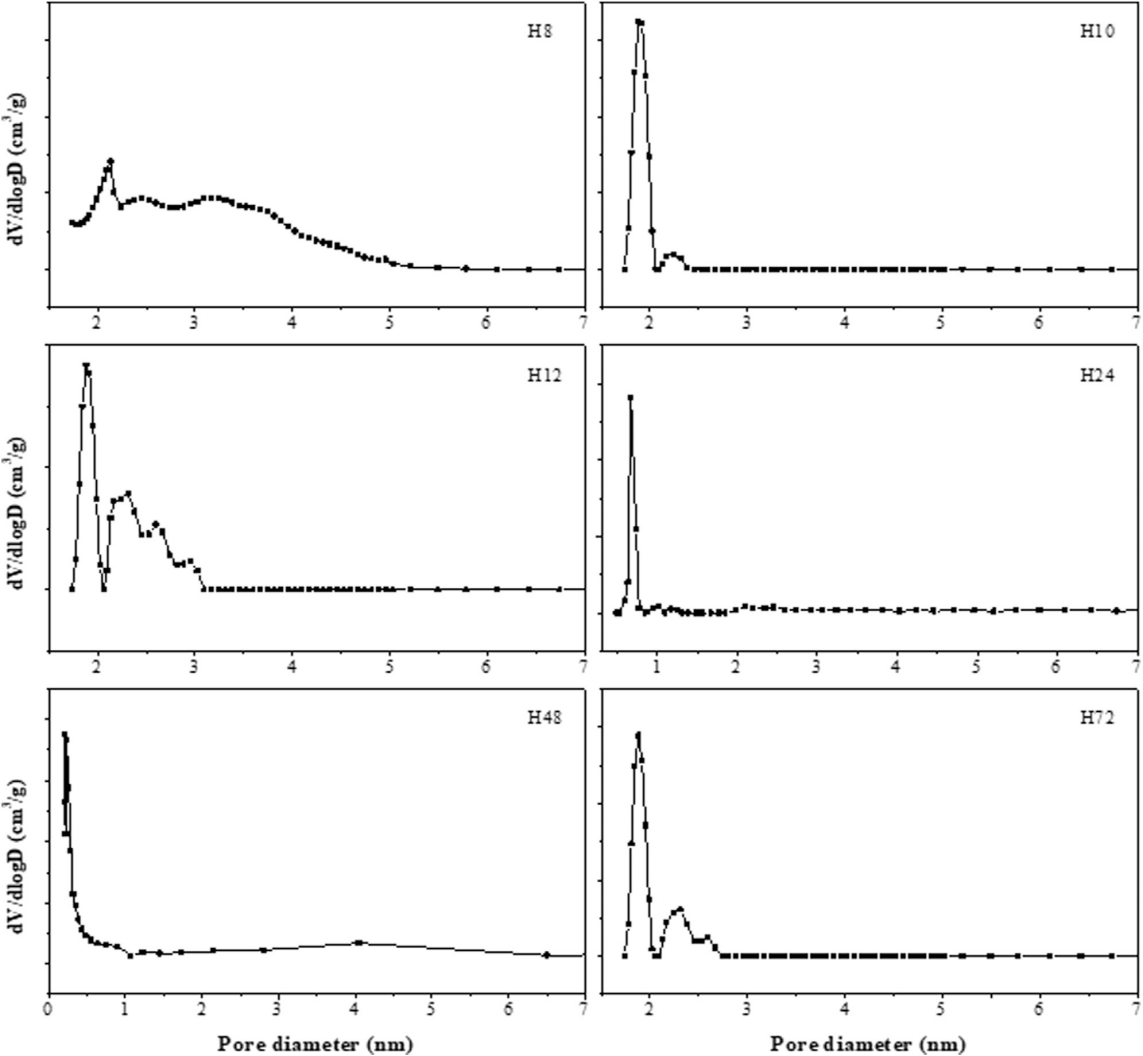
Mesopore size distributions of as-synthesized HBEA zeolites produced from CFA nanosilica extract at different hydrothermal times.

The deduced density functional theory (DFT) pore size distribution of HBEA zeolites showed mesopore size distribution curves above 1 nm (Figure 9), with samples prepared hydrothermally for 8, 10, and 12 h (H8, H10, and H12). In the case of H8, a major peak corresponding to a typical zeolitic mesoporous structure (361 m^2^/g) was observed at about 2.1 nm. The extension of minor and poorly defined peaks observed within the mesoporous region of 2.4 and 5 nm confirmed the presence of disordered mesopores, as indicated by TEM (Figures 5A,B). Also, this may suggest an incomplete condensation of some aggregates at the short synthesis time, which can be linked to reduced peak intensity, as observed in the XRD pattern (Figure 2-H8). Samples H10 and H12, with the same molar water fraction of 3.99 but different synthesis durations of 10 and 12 h, respectively, showed a strong mesopore contribution centered around 2 nm. It is noteworthy that a well-defined peak around 2.2 nm was found in sample H10, while as the hydrothermal time increased to 12 h (H12), a multimodal pore distribution with several peaks within the range of 2–3 nm was shown (Figure 9). Hence, the well-defined pore size distribution within 2–4 nm can be related to the high mesoporous surface area of 488 and 425 m^2^/g of samples H10 and H12, respectively.

With longer hydrothermal synthesis duration, as presented in Figure 9, mesopore size diminished and two minor peaks at 1 and 1.25 nm (H24) were detected, these being associated with the presence of significant microporous surface area (validated by the micropore area, 512 m^2^/g in Table 2). Interestingly, a significantly higher mesopore size was observed in the H72 zeolite, with a major peak at 1.9 and a minor broad peak at 2.3 nm. This observation further supports the increased surface area, micropore area, and mesopore area shown in the BET results (Table 2). Indicatively, aside from the microporosity in HBEA, a secondary porosity has been formed, thus confirming the hierarchical porous structure. Hence, the synthesized HBEA zeolite samples with poorly defined peaks within the mesopore range indicated the presence of defects alongside porosity. Meanwhile, the broad pore distribution within the range of 2–5 nm confirms the mesoporosity of the zeolite and the hierarchical pore distribution (Escola et al., 2018).

**FIGURE 10:**
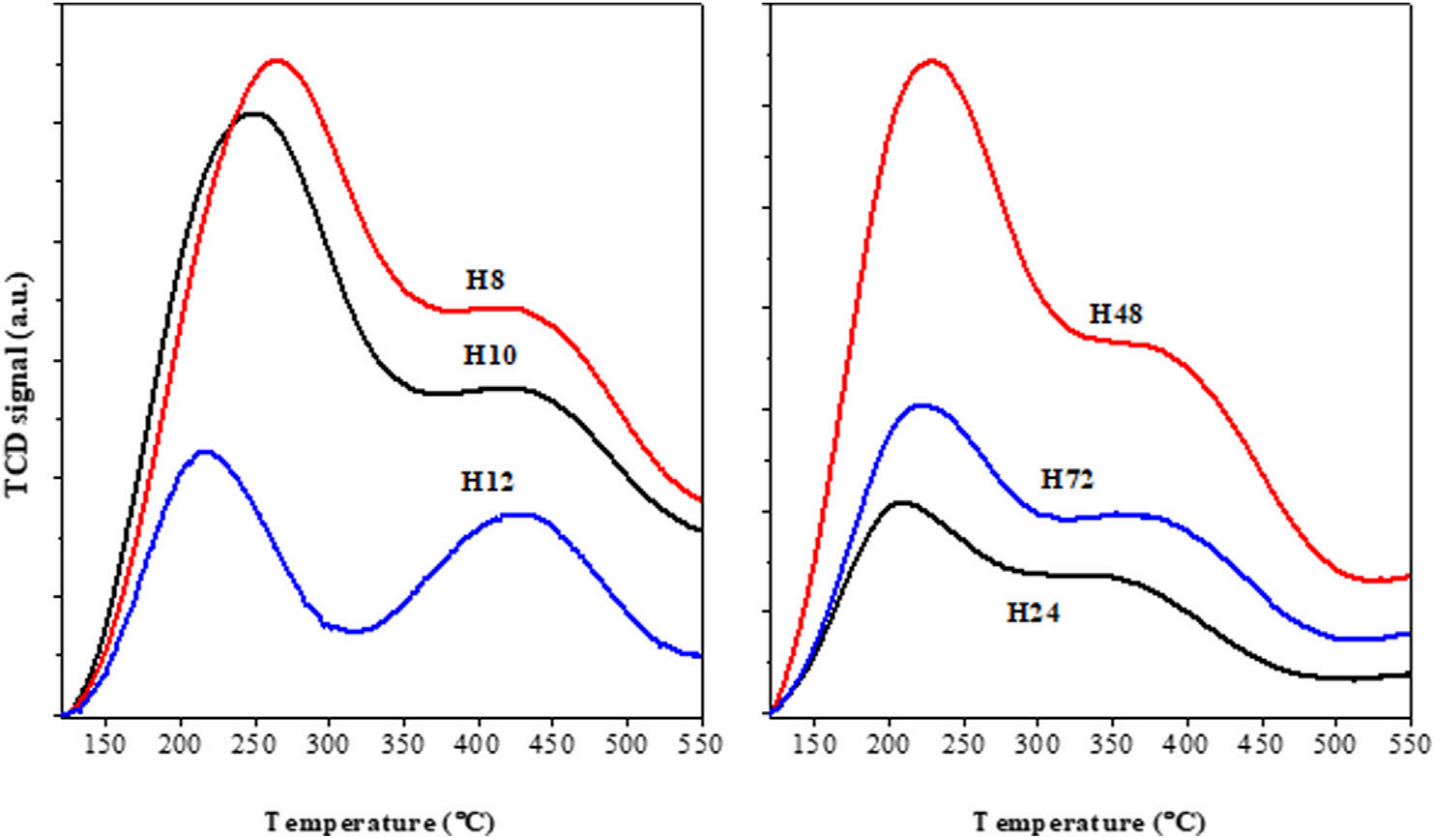
TPD profiles of as-synthesized HBEA zeolites produced from CFA nanosilica extract at different hydrothermal times.

The presence of acid sites in HBEA zeolite samples was assessed via NH_3_ TPD experiments, as shown in Figure 10. Herein, all HBEA samples exhibited two distinctive NH_3_ desorption peaks at low (212–253°C) and high (365–451°C) temperatures, which are consistent with the presence of weak acid and strong acid sites, respectively. Generally, the hydrogen-bonded NH_3_ molecules correspond to the weak acid sites. On the other hand, chemisorbed NH_3_ molecules correspond to strong acid sites, which are associated with the framework aluminum atoms (Wang et al., 2017; Monama et al., 2020).

Table 3 presents the detailed acid strength of the HBEA zeolites at the different desorption temperature regions.

**TABLE 3:** Acidity determined by NH_3_ TPD of as-synthesized HBEA zeolites.

Sample	Acidic strength				Total acid sites (mmol/g)	NMR Si/Al ratio	EDS Si/Al ratio
	Weak (mmol/g)	T_1_ (°C)	Strong (mmol/g)	T_2_ (°C)			
H8	2.39	236	0.26	447	2.65	29.7	30
H10	2.46	253	0.29	451	2.75	26.8	25
H12	0.90	212	0.66	428	1.56	30.0	25
H24	0.63	203	0.17	365	0.80	24.8	24
H48	1.55	217	0.20	402	1.75	23.7	24
H72	0.58	213	0.14	392	0.72	22.9	23

The amounts of NH_3_ desorbed at low temperatures (212–253°C) were between 0.6 and 2.5 mmol/g NH_3_, as shown in Table 3. The maximum adsorption of NH_3_ due to the weak acid sites (2.39 and 2.46 mmol/g) was observed in samples H8 and H10. By increasing the hydrothermal treatment duration, the peak of the weak acidity shifted to a lower temperature, and the NH_3_ adsorption declined to a minimum between 0.58 and 1.55 mmol/g. The strong acid sites of the CFA-based HBEA zeolites can be grouped into high, 0.66 mmol/g (H12), mid-high, 0.17–0.29 mmol/g (H24, H48, H10, and H8), and low, 0.14 mmol/g (H72).

### Acylation of Anisole With Benzoyl Chloride Over HBEA Zeolites

**FIGURE 11:**
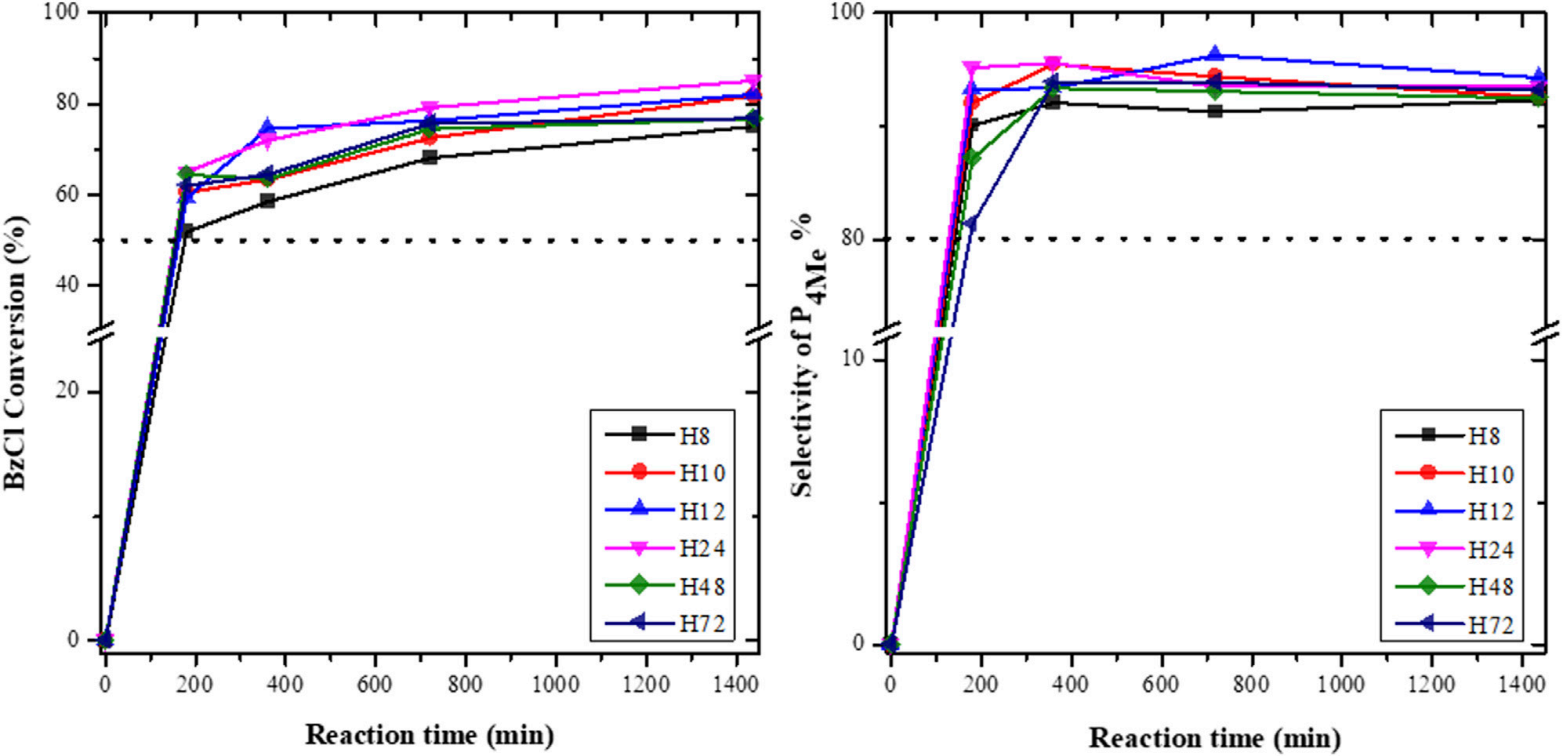
BzCl conversion and selectivity toward P_4Me_ over diferent CFA-based HBEA zeolites (reaction conditions: temperature = 120°C, maximum time = 1,440 min, anisole/benzoyl chloride ratio = 2.5:1, and catalyst = 0.068 g).

The catalytic activities of the H8, H10, H12, H24, H48, and H72 zeolites were evaluated in the acylation of anisole with BzCl (Figure 11). It is noteworthy that the blank reaction without catalysts did not result in the formation of any product.

Figure 11 depicts the conversion of anisole over HBEA catalysts. The total conversion of BzCl with anisole over all HBEA zeolite catalysts was between 50 and 83%, which was predominantly >50% of the *p*-position (P_4Me_) and <35% of the other products (*o*-position (P_2Me_) and phenyl benzoate). Inductively, this shows that all CFA-based HBEA zeolites were active catalysts for this acylation reaction. This is similar to HBEA, MOR, and Al-SBA-15 catalysts synthesized using commercial feedstock raw chemicals, which resulted in the formation of the same three products of *p*- and *o*-position and phenyl benzoate in the acylation of BzCl with anisole. Also, the *p*-position of 4-methoxyacetophenone (P_4Me_) was the major product formed in the conversion of anisole over Al-SBA-15 and HBEA zeolites (El Berrichi et al., 2005; Wagholikar et al., 2007). Therefore, the CFA-based HBEA zeolites demonstrated similar catalytic activity to that of conventional benchmark HBEA zeolites.

**FIGURE 12:**
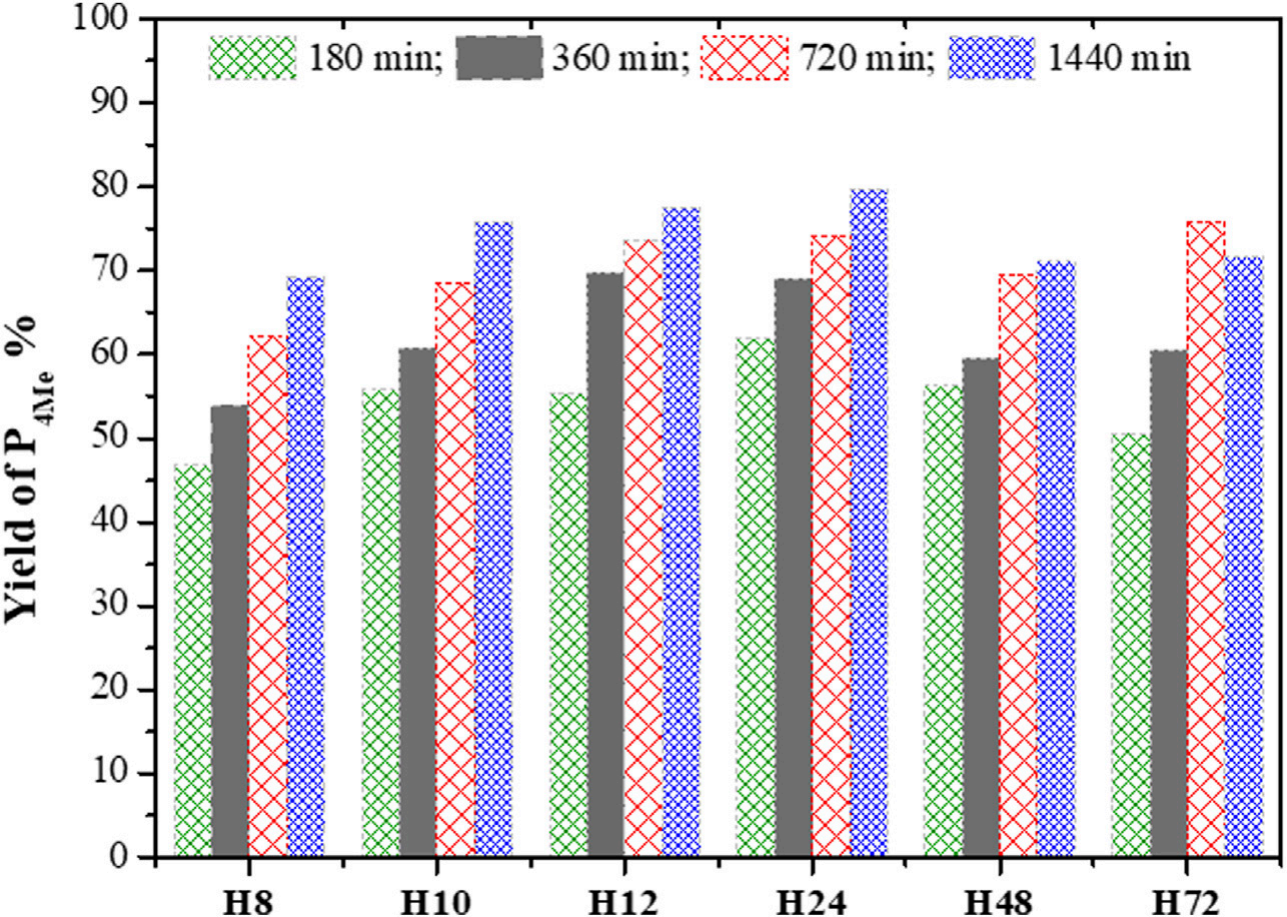
Percentage yield of P_4Me_ over CFA-based HBEA zeolites (reaction conditions: temperature = 120°C, maximum time = 1,440 min, anisole/BzCl ratio = 2.5:1, and catalyst = 0.068 g).

Notably, a steadily increasing conversion as a function of the reaction time was observed in Figure 11. To substantiate the aforementioned results, the selectivity of P_4Me_ and the product yield over as-synthesized HBEA zeolite catalysts are presented in Figures 11, 12, respectively.

The selectivity in P_4Me_ over as-synthesized HBEA zeolites was maintained between 80> P_4Me_ ≤96%, with the highest selectivity of 96% recorded for samples H12 and H24 (Figure 11). After a reaction time of 360 min, the selectivity of most samples declined slightly by 1–3%, which was then maintained with a prolonged reaction time. Meanwhile, the reduced selectivity of H12 by 2% occurred at a reaction time of 720 min. This suggests that the catalytic performance of CFA-based HBEA zeolites may be influenced by one or two or a combination of the following factors: surface area, mesoporous area, pore size distribution (porosity), and framework aluminum structure.

The percentage yield of P_4Me_ over the different HBEA zeolite samples was compared as presented in Figure 12. It was found that an increased yield of P_4Me_ is also a function of increased acylation duration. The gradual yield increase of P_4Me_ with the reaction time was maintained between 47 and 80% over CFA-based HBEA zeolites. Consequently, the highest P_4Me_ yield mostly achieved at all reaction times was observed over the H12 and H24 zeolites. Hence, this suggests that the *p*-methoxyacetophenone (P_4Me_) selectivity and yield were enhanced over the porous structure and the well-distributed mesoporous structure of HBEA zeolites (Figure 9; Table 2).

To this end, the catalytic performance of H12 and H24 with increased acylation time correlated with the presence of strong framework Al (Supplementary Table S2). The strong FAl presence of 45% associated with both H12 and H24 was observed to influence the yield of P_4Me_ with a progressive and constant percentage increase of 55<70<74<78% and 62<69<74<80% with increased reaction time, respectively. Since the FAl can be associated with acid sites, this suggests that the acidity of the zeolite catalyst influenced the degree of conversion. This shows that with a reduced amount of EFAl species, the acid site of the framework aluminum is more exposed, thus allowing high interaction between the molecules, thereby shifting the equilibrium toward the designed products (Aleixo et al., 2017). The preferential selectivity of the *p*-position and the limitation suffered by the *o*-position could be associated with strong steric hindrance in the zeolite pores (Kim et al., 2015; Wang et al., 2017).

Furthermore, the high selectivity of P_4Me_ over the H12 and H24 zeolites is probably due to the high mesoporosity and pore distribution in the catalyst, allowing easier diffusional pathways of the reactants within the mesopores of a well-distributed hierarchical pore–structured zeolite. The well-defined mesopore structure between 2 and 3.5 nm in samples with high mesopore areas of 208 and 210 m^2^/g allowed better contact of the reactant with the acid sites (Figures 9, 10; Table 2). Thus, the well-defined hierarchical porosity of H12 and H24 favored better diffusion and limited steric hindrance in the zeolite pores. Therefore, the large mesoporous area allows accessibility of the diffused reactants to interact with numerous acid sites and facilitates the selectivity of zeolites in the acylation reaction (Serrano et al., 2009; Kim et al., 2015).

The efficiency of the catalytic conversion of anisole and the selectivity of P_4Me_ over fly ash–based HBEA zeolites are a function of reaction time, framework Al, surface area, large mesopore area, and active acid sites. Hence, this study demonstrated that the catalytic performance of rapidly synthesized CFA-based HBEA zeolites is equivalent to that of pure chemical-based synthesized HBEA zeolites.

## Conclusion

The synthesis of HBEA zeolites from CFA at a short hydrothermal time of 8–12 h was a function of the reduced molar fraction of water (1.776≤ x ≤3.991) in the applied molar regime (1Si: 0.017Al: 0.241Na: 0.399TEAOH: *x*H_2_O). High molar water fraction hinders the complete formation of the pure BEA zeolite phase within a short hydrothermal synthesis time. The minimum molar water fraction of 1.77 induced rapid crystallization of HBEA zeolites within the shortest hydrothermal time of 8 h (or even less). The rapidly grown HBEA crystals exhibited a porous structure with significant surface area, high mesopore area, and strong acid sites.

The synthesized CFA-based HBEA zeolite was highly active in the acylation of anisole with benzoyl chloride. A gradual increase in the conversion with time was observed. Rapidly grown zeolite catalysts at hydrothermal times of 12 and 24 h led to the highest catalytic performance with a significantly increased selectivity of 96% (corresponding to 75–80% yield) in 4-methoxyacetophenone. The different factors that influenced the performance of the CFA-synthesized HBEA zeolite in the acylation of anisole with BzCl included framework aluminum, surface area, large mesopore area, and active acid sites. Therefore, the HBEA zeolites appear to be a suitable heterogeneous catalyst for liquid-phase Friedel–Crafts reactions.

## Data Availability

The original contributions presented in the study are included in the article/Supplementary Material; further inquiries can be directed to the corresponding author.
