# Supplementary material for: Acylation of Anisole With Benzoyl Chloride Over Rapidly Synthesized Fly Ash–Based HBEA Zeolite
Source: Front Chem. 2021 Jun 18;9:683125. doi: 10.3389/fchem.2021.683125 (PMC8249572; doi:10.3389/fchem.2021.683125)
Supplement: Supplementary file 1 [file Presentation1.pdf]

Supplementary material

Acylation of Anisole With Benzoyl Chloride Over Rapidly Synthesized Fly Ash–Based HBEA Zeolite

Alechine E. Ameh[1]*, Nicholas M. Musyoka[2], Oluwaseun Oyekola[3], Benoit Louis[4] and Leslie F. Petrik[1]

[1]Environmental and Nano Science Research Group, Department of Chemistry, University of the Western Cape, Bellville, South Africa, [2]Centre for Nanostructures and Advanced Materials (CeNAM), Chemicals Cluster, Council for Scientific and Industrial Research (CSIR), Pretoria, South Africa, [3]Department of Chemical Engineering, Cape Peninsula University of Technology, Cape Town, South Africa, [4]Institut de Chimie et Procédés pour l'Energie l'Environnement et la Santé (ICPEES), UMR 7515, CNRS, Université de Strasbourg, Strasbourg, France

Supplementary Table S1. Molar composition for the synthesis mixture and phase formation at applied conditions

**Code name**	[a]**Molar ratio of synthesis mixture**					**Synthesis time, h**	[b]**Phase formation**
	Si	Al	Na	TEAOH	H_2O		
H01	1	0.017	0.241	0.399	17.958	72	AMR
H02	1	0.017	0.241	0.399	12.828	72	AMR+BEA
H72	1	0.017	0.241	0.399	8.980	72	BEA
H48	1	0.017	0.241	0.399	8.980	48	BEA
H24	1	0.017	0.241	0.399	8.980	24	BEA
H12	1	0.017	0.241	0.399	8.980	12	AMR
C11	1	0.017	0.241	0.399	5.986	12	AMR
C12	1	0.017	0.241	0.399	3.991	12	BEA
C13	1	0.017	0.241	0.399	2.661	12	BEA
C14	1	0.017	0.241	0.399	1.776	12	BEA
B09	1	0.017	0.241	0.399	3.991	10	BEA
B10	1	0.017	0.241	0.399	2.661	10	BEA
B11	1	0.017	0.241	0.399	1.776	10	BEA
A7	1	0.017	0.241	0.399	3.991	8	BEA
A8	1	0.017	0.241	0.399	2.661	8	BEA
A9	1	0.017	0.241	0.399	1.776	8	BEA

[a]Calculated from the metal oxide as determined by XRF of the nanosilica and the amount of NaOH or Al added.
[b]AMR means an amorphous phase in the as-synthesised product as determined by XRD.

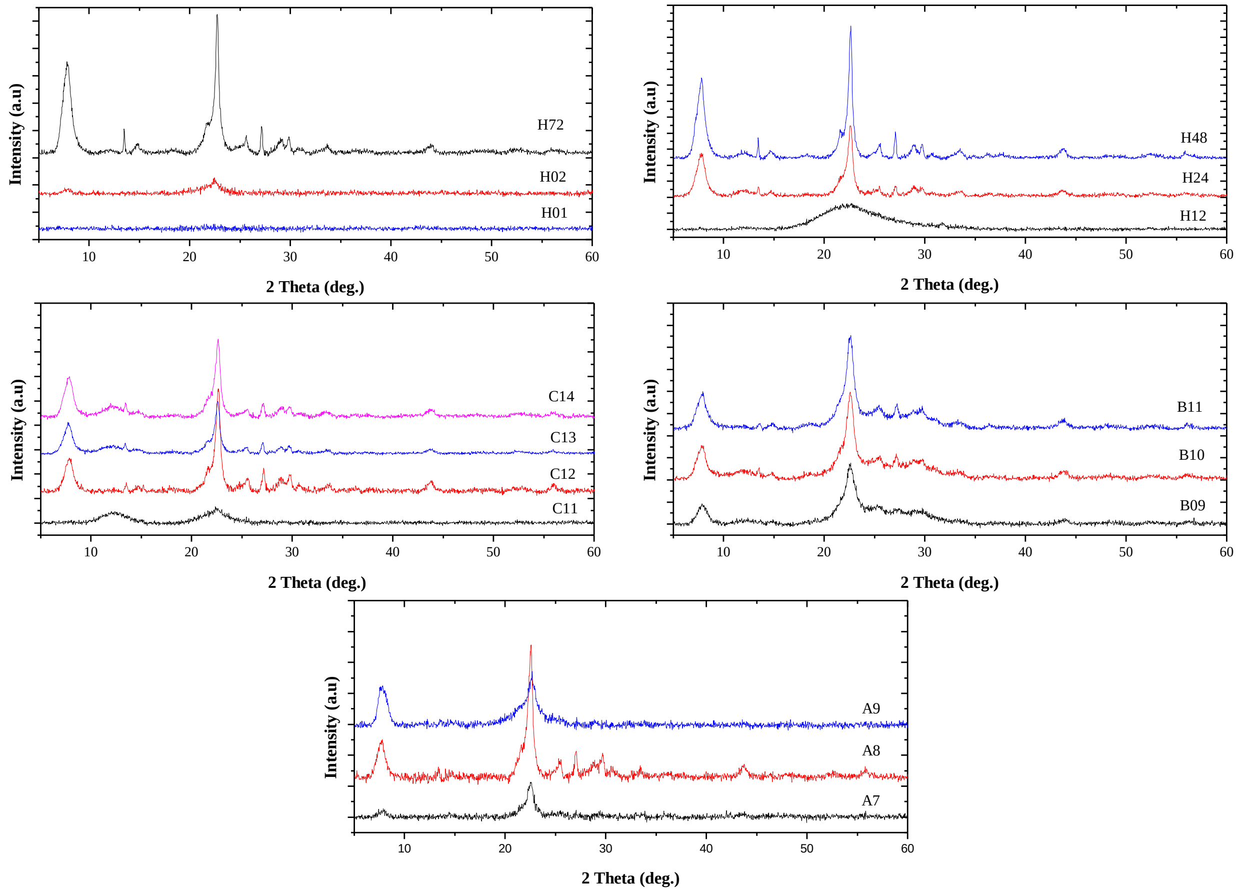

Supplementary Figure S1. XRD patterns showing the effect of reduced molar quantities of water in the synthesis mixture on the hydrothermal time and phase purity of BEA zeolites (see Table 1).

Supplementary Table S2. Detailed ^{27}Al MAS NMR spectroscopic data after FAl and EFAl deconvolution in HBEA samples.

Sample	Framework Al					Extra-framework Al		
	$^{1}T_{Al}$	Integral (%)	$^{2}T_{Al}$	Integral (%)	T_{Al} total	$^{3}T_{Al}$	Integral (%)	Width (ppm)
H8	57.9	36.6	54.4	42.6	79.2	0.4	20.8	1.8
H10	57.9	42.5	54.5	31.2	73.7	0.5	26.3	1.9
H12	57.9	31.3	54.4	44.6	75.9	0.4	24.1	2.1
H24	57.5	42.4	54.0	44.7	87.1	0.3	12.9	1.7
H48	60.9	51.1	56.6	16.5	67.5	3.0	32.5	1.4
H72	57.5	35.4	53.8	53.9	89.3	0.3	10.7	1.5

$^{1}T_{Al}$, $^{2}T_{Al}$ and $^{3}T_{Al}$ is the concentration of FAl and EFAL peaks in ppm

T_{Al} is total % integral of $^{1}T_{Al}$ and $^{2}T_{Al}$

Supplementary Table S3. Chemical shift, Si/Al ratio, intensity and area of deconvoluted Q^3 and Q^4 peak.

Samples	Q^3 Si(1Al)		Q^4 Si(0Al)		NMR	EDS
	δ_{decon} (ppm)	Area %	δ_{decon} (ppm)	Area %	Si/Al ratio	Si/Al ratio
H8	-102.9	23.7	-111.0	41.1	29.7	30
H10	-104.4	25.1	-111.1	43.4	26.8	25
H12	-104.2	22.8	-111.3	45.5	30.0	25
H24	-103.3	24.6	-110.7	45.8	24.8	24
H48	-103.3	27.1	-110.9	38.9	23.7	26
H72	-103.1	26.3	-110.8	42.9	22.9	23

δ_{decon} determined from NMR chemical shifts.

The derived framework Si/Al ratio was calculated according to equation below (Holzinger et al., 2018).

$$\frac{Si}{Al}(^{29}\text{Si}) = \frac{2.I[Q^2\{2Al\}]+3.I[Q^3\{1Al\}]+4.I[Q^4\{0Al\}]}{I[Q^3\{1Al\}]}$$

where I is the peak intensity of Q^2, Q^3 and Q^4 resonances identified by deconvolution of the ^{29}Si MAS NMR spectra.

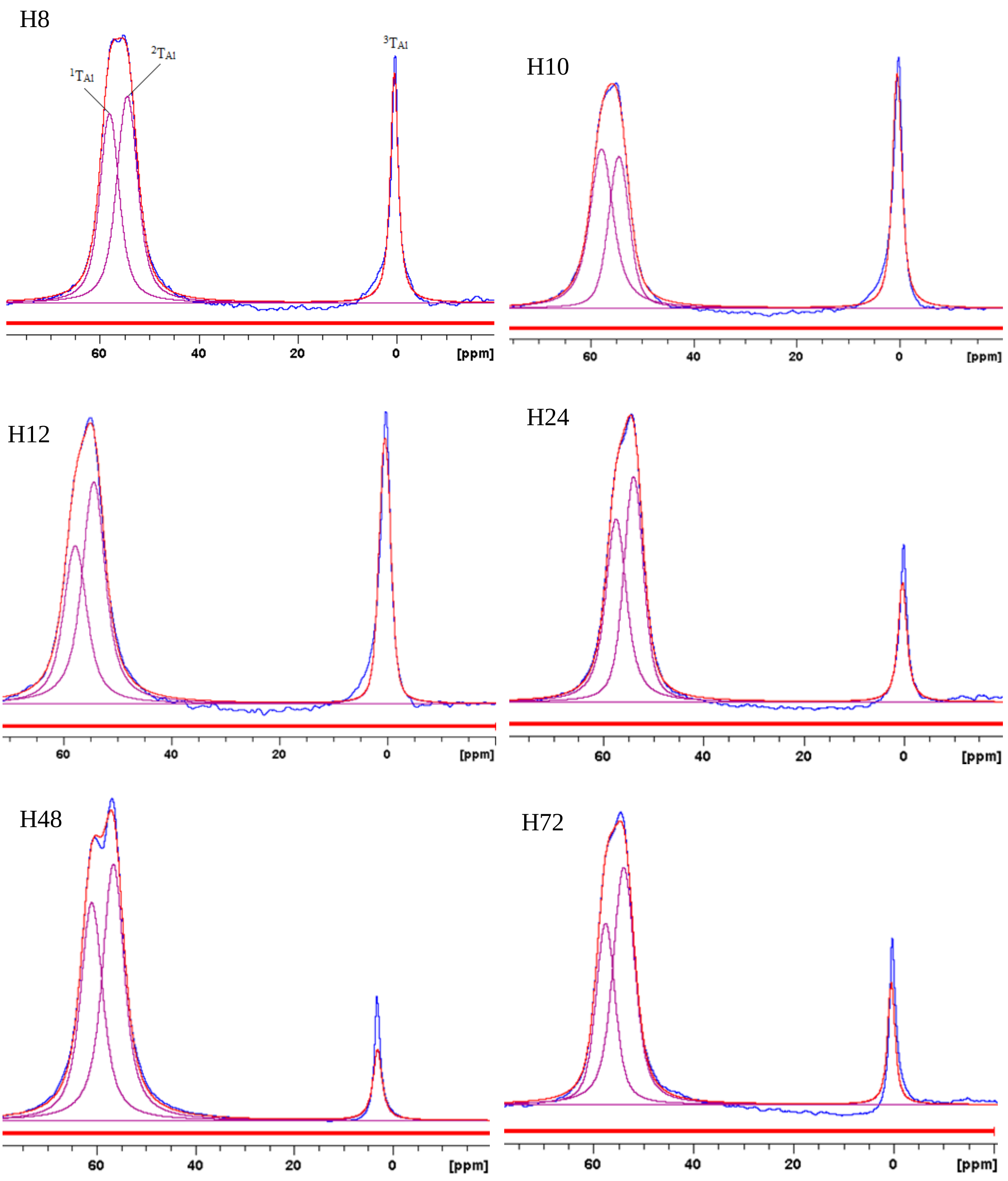

Supplementary Figure S2. Deconvoluted ^{27}Al MAS NMR spectra of HBEA zeolites.

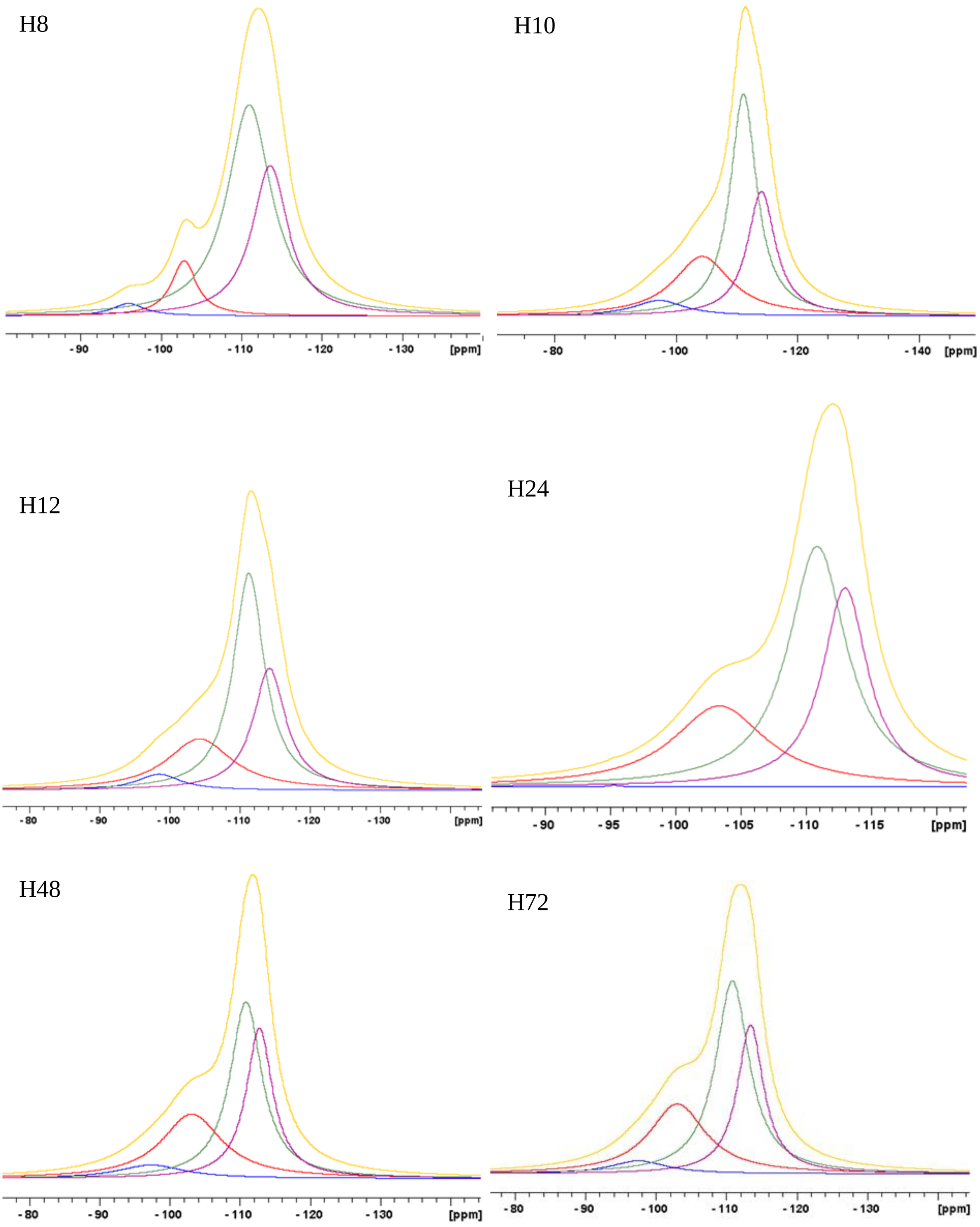

Supplementary Figure S3. Deconvoluted ^{29}Si MAS NMR spectra of HBEA zeolites.

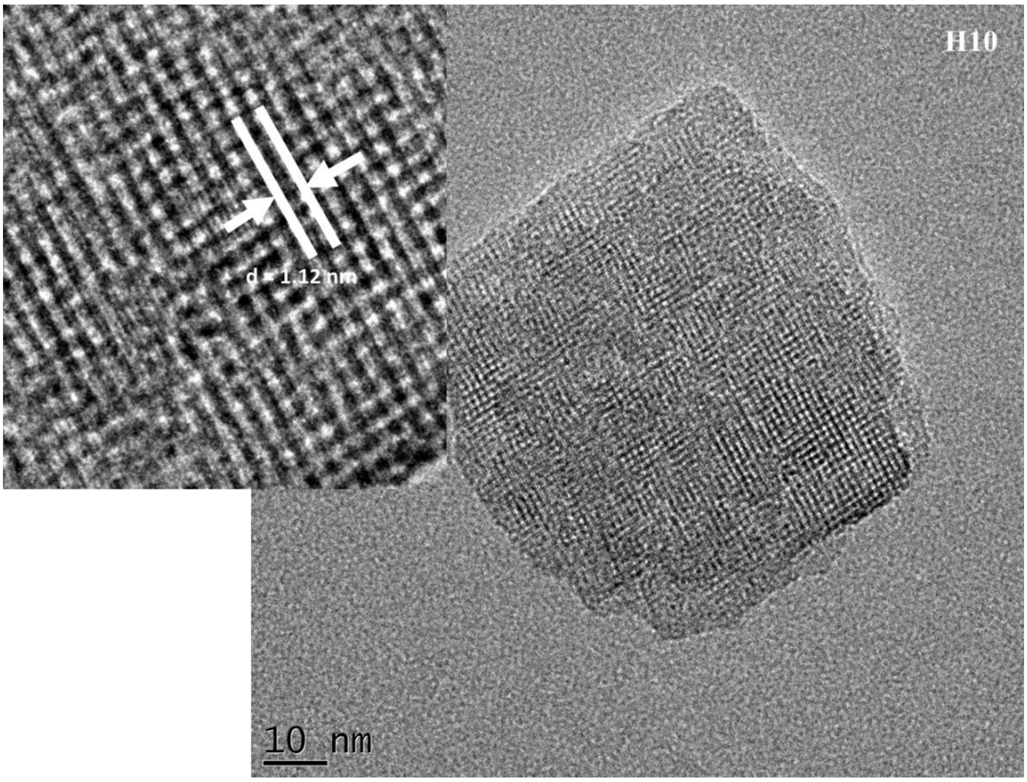
H10
d = 1.12 nm
10 nm

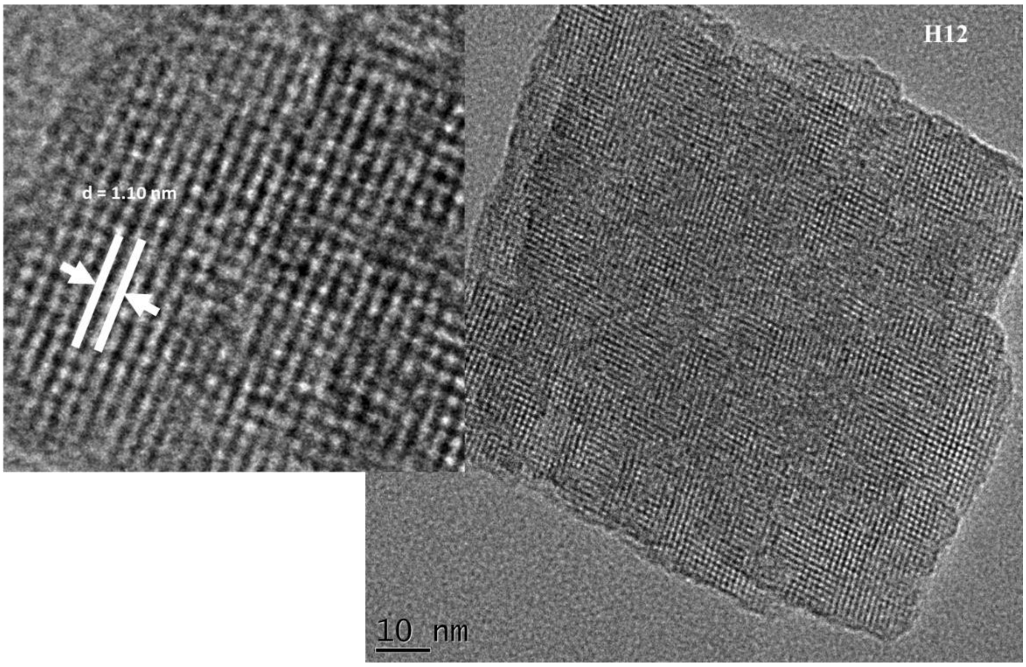
H12
d = 1.10 nm
10 nm

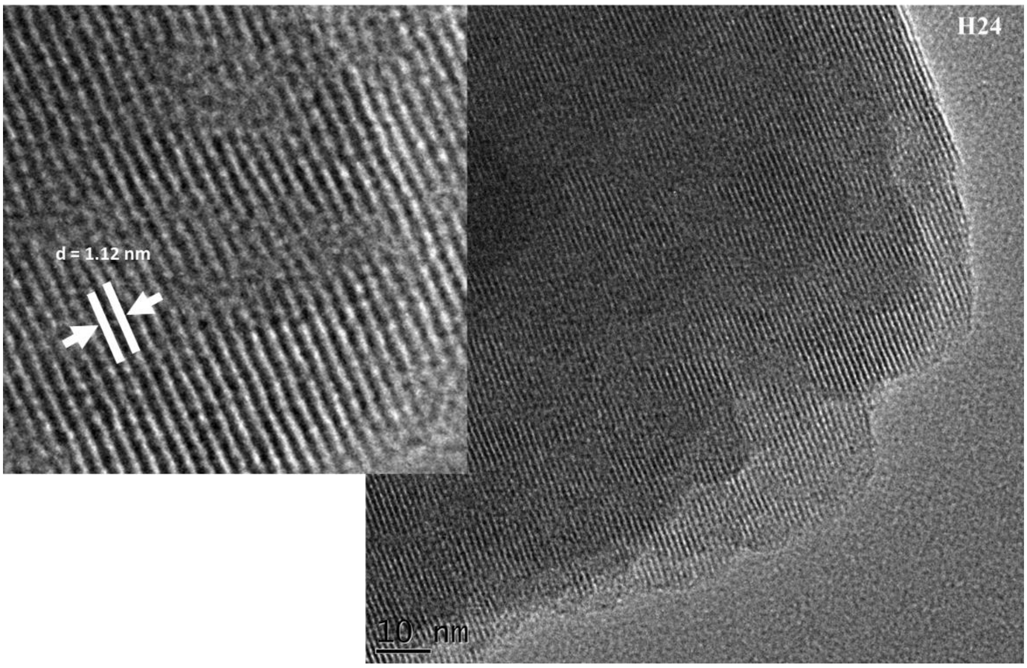

Supplementary Figure S4. TEM images of as-synthesised HBEA zeolites indicating the d-space.

References:

Holzinger, Julian, Pablo Beato, Lars Fahl Lundegaard, and Jørgen Skibsted. "Distribution of aluminum over the tetrahedral sites in ZSM-5 zeolites and their evolution after steam treatment." The Journal of Physical Chemistry C 122, no. 27 (2018): 15595-15613.
